# Use of sacubitril/valsartan in Marfan syndrome–related cardiomyopathy: The first case report

**DOI:** 10.1097/MD.0000000000017978

**Published:** 2019-11-22

**Authors:** Silvia Spoto, Emanuele Valeriani, Luciana Locorriere, Giuseppina Beretta Anguissola, Angelo Lauria Pantano, Francesca Terracciani, Maria Caterina Bono, Sebastiano Costantino, Massimo Ciccozzi, Silvia Angeletti

**Affiliations:** a Internal Medicine Department, University Campus Bio-Medico of Rome, Rome; b Internal Medicine Department, University G. D’Annunzio, Chieti; c Cardiology Department; d Unit of Medical Statistic and Molecular Epidemiology; e Unit of Clinical Laboratory Science, University Campus Bio-Medico of Rome, Rome, Italy.

**Keywords:** heart failure, Marfan syndrome, midregional-proadrenomedullin, N-terminal pro-brain natriuretic peptide, sacubitril-valsartan

## Abstract

**Rationale:**

Marfan syndrome is a rare cause of heart failure due to primary or secondary cardiomyopathy. Recently, sacubitril/valsartan—an angiotensin receptor blocker-neprilysin inhibitor—has been added in clinical practice as a standard therapy for heart failure. To our knowledge, there are no data on sacubitril/valsartan's effects on cardiovascular outcomes in patients with Marfan syndrome.

**Patient concerns:**

A 24-year-old man was admitted to our Internal Medicine Department due to dyspnea, ascites, and leg swelling. Arterial blood gas analysis revealed severe hypoxemia with respiratory and metabolic alkalosis. Hilar congestion was highlighted on chest x-ray.

**Diagnoses:**

Recurrent acute decompensated heart failure with reduced ejection fraction despite optimal medical therapy in Marfan-related cardiomyopathy.

**Interventions and outcomes:**

Sacubitril/valsartan was added to optimal medical therapy after hemodynamic stabilization allowing progressive clinical, laboratoristic, and echocardiographic improvement. Patient maintained a free survival from heart failure and a good quality of life until 9-month follow-up.

**Lessons:**

Sacubitril/valsartan should be effective on pathophysiologic mechanisms and cardiovascular outcomes of Marfan syndrome–related cardiovascular complications.

## 1 Introduction

Heart failure (HF) is infrequent in young people showing various etiologies within different world regions. Marfan syndrome (MFS) can be listed as a rare cause of HF.

MFS is a connective-tissue autosomal dominant inherited disorder characterized by 400 individual mutations in the gene of fibrillin-1 with a prevalence of 1.5 to 17.2 per 100,000 person in the general population.^[1,2]^ MFS diagnosis is made by the use of 2010 revised Ghent-2 nosology criteria based on the presence of family history along with other specific diagnostic criteria.^[1,3]^ Single- and multi-organ involvement (ocular, skeletal, and cardiovascular system) and absence of a curative therapy are responsible for the chronic, severe, and life-threatening course of the disease.^[1]^ Nevertheless, amelioration in medical and surgical management produced an increase in life expectancy from 32 to >50 years.^[4]^

Patients with MFS present primary cardiomyopathy for heart muscle fibrillin-1 deficiency in 3.08% of cases (left ventricular dysfunction due to hypertrophy and dilatation, abnormal chordae tendineae with mitral valve prolapse and regurgitation, and increased prevalence of Wolff-Parkinson-White syndrome) and/or cardiac involvement secondary to ischemic complication of cardiovascular surgery in 8.07% of cases.^[4]^ Furthermore, clinically relevant cardiovascular manifestations consisting in root and descending-aorta dissection and rupture due to progressive aortic dilatation represent the main cause of mortality.^[3]^

Recently, the angiotensin receptor blocker-neprilysin inhibitor sacubitril/valsartan has been introduced in clinical practice as treatment for HF showing its superiority to ACE inhibition alone in reducing the risks of death and hospitalization.^[5–9]^ Indeed, sacubitril/valsartan influences the 2 HF pathophysiological mechanisms. While valsartan inhibits renin-angiotensin-aldosterone system (RAAS) through angiotensin II type-1 (AT1) receptor blockage, sacubitril inhibits neprilysin and increase natriuretic peptides-atrial natriuretic peptide, brain natriuretic peptide (BNP), C-type natriuretic peptide, bradykinin, and adrenomedullin values.^[10]^

We report a case of a III–IV New York Heart Association (NYHA) class, stage C reduced ejection fraction HF in young patient with MFS occurred despite optimal medical therapy and successfully treated with sacubitril/valsartan once reached persistent hemodynamic stabilization.

## 2 Case presentation

A 24-year-old man with MFS-related cardiovascular complications was admitted to our Internal Medicine Department because of 3 consecutive episodes of acute decompensated HF with reduced ejection fraction (ADHFrEF) from 2014 through 2017. His remaining medical history includes arterial hypertension, dyslipidemia, hyperthyroidism, anaphylactic shock due to flecainide, and previous tabagic habit.

MFS diagnosis was made in 2010 when ascending aorta and aortic valve substitution with mechanical prosthesis were performed due to type A acute aortic dissection in presence of family history—mother, father, and 3 sons with MFS. During intraoperative period, inferior acute coronary syndrome occurred and was treated with coronary artery bypass graft surgery.

On May 2014, mechanical descending aorta and aortic arch prosthesis implantation were performed due to chronic dissecting aneurysm. Peri-aortic hematoma and medullary ischemia complicated surgery and caused lower limb paraplegia, dolorific and thermal hypoesthesia, and rectal incontinence. After 2 months, during his first admission for ADHFrEF, laboratory tests were notable for N-terminal pro-BNP (NT-proBNP) of 12,000 pg/mL and transthoracic echocardiogram showed normal prosthesis function, dilatation of all cardiac chambers (mainly the left ones) with moderate mitral and tricuspid insufficiency, left ventricular hypertrophy, inferior wall akinesis, septal dyskinesis, and hypokinesis of the remaining walls with severe left ventricular ejection fraction (LVEF) reduction −20%. He was treated with intravenous diuretics and internal cardiac defibrillator (St. Jude Ellipse DR, DDD mode) without cardiac resynchronization therapy (electrocardiogram criteria not met) and discharged on maximal medical therapy (furosemide, carvedilol, ramipril, spironolactone, digoxin, and amlodipine).

New episode of ADHFrEF occurred on august 2017. Notable admission laboratoristic values included NT-proBNP of 2719 pg/mL and midregional-proadrenomedullin (MR-proADM) of 1.61 nmol/L, whereas transthoracic and transesophageal echocardiogram showed an LVEF of 35%, normal prosthesis function, severe tricuspid regurgitation, and rupture of the anterior leaflet of the chordae tendineae with severe mitral regurgitation. Patients were treated yet with intravenous diuretics and digoxin and discharged on optimal medical therapy (furosemide, carvedilol, ramipril, spironolactone, digoxin, and amlodipine).

Lastly, he was readmitted to our Internal Medicine Department on November 2017 due to third episode of ADHFrEF and infected mediastinal fluid collection secondary to 1 month before severe valvular insufficiency surgical correction with mitral mechanical prosthesis implantation (31-mm ST Jude) and De Vega tricuspid annuloplasty.

Physical examination was notable for blood pressure 120/80 mm Hg, pulse 82 bpm, respiratory rate 26 apm, O_2_ saturation 87% on room air with orthopneic obligatory position, and Marfanoid habitus (weight 147 kg, height 2.2 m). Cardiopulmonary evaluation revealed metallic second heart sound, stony dull percussion with reduced tactile vocal fremitus and crackles at the basis of the lungs, widely diminished vesicular breath sounds, presence of abundant ascites, and leg swelling.

Notable laboratoristic values included NT-proBNP of 10,132 pg/mL and MR-proADM of 2.36 nmoL/mL.

**Figure 1:**
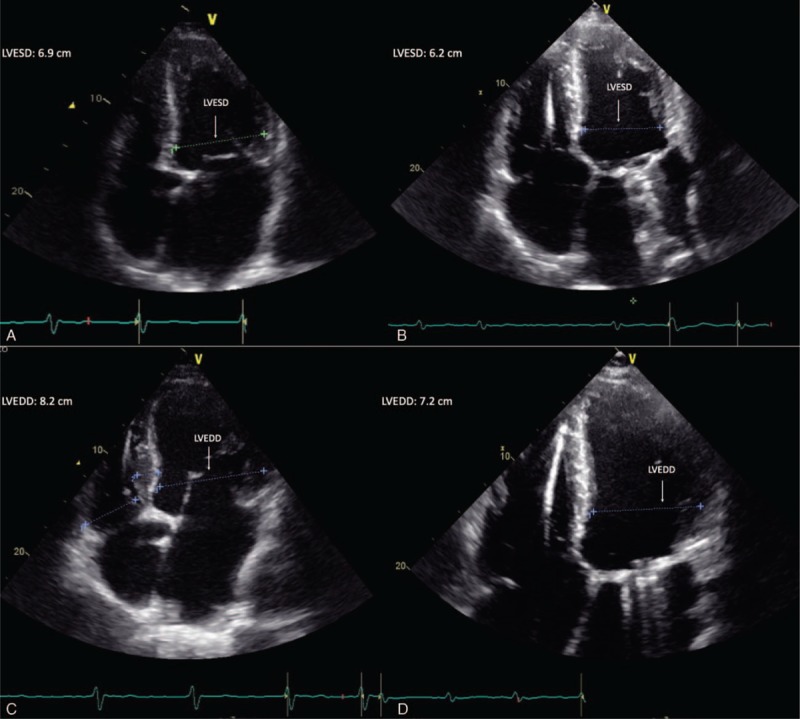
Echocardiographic features before (A, C) and after (B, D) 9 months of sacubitril/valsartan administration. LVEDD = left ventricle end-diastolic diameter, LVESD = left ventricle end-systolic diameter.

Arterial blood gas analysis revealed severe hypoxemia with respiratory and metabolic alkalosis (pH 7.56, pO_2_ 46 mm Hg, pCO_2_ 24.8 mm Hg, HCO^3−^ 21.9 mmol/L, alveolar-arterial gradient 31 mm Hg). Although electrocardiogram highlighted atrial fibrillation with ventricular rate of 80 bpm, left ventricular hypertrophy, and inferolateral subepicardial ischemia, transthoracic echocardiogram showed the same features as the previous except for LVEF of 30%, presence of mitral mechanical prosthesis, with paravalvular leak and mean gradient of 9 mm Hg, and mild tricuspid insufficiency (Fig. 1 A and C).

Hilar congestion with cardiomegaly, right upper lobe pulmonary consolidation, and infected mediastinal fluid collection have been shown on chest x-ray and high-resolution computed tomography, respectively.

Patient was treated with intravenous furosemide 250 mg q.d., canrenone 100 mg q.d., piperacillin/tazobactam 4.5 g q6h, and teicoplanin 12 mg/kg b.i.d., then 12 mg/kg q.d. On day 9, once reaching hemodynamic stabilization, sacubitril/valsartan midrange dose of 49/51 mg b.i.d. has been added to carvedilol 3.125 mg b.i.d., spironolactone 100 mg q.d., furosemide 250 mg q.d., and digoxin 0.25 mg q.d.

On 1-month follow-up sacubitril/valsartan has been increased to 97/103 mg b.i.d. for patient's good clinical condition, allowing persistent and progressive clinical, laboratory (NT-proBNP reduction and MR-proADM increase), echocardiographic-LVEF increase (42% vs 30%), left ventricle end-systolic diameter, left ventricle end-diastolic diameter, left ventricular mass, and left ventricular mass index reduction, and quality of life improvement without new episodes of ADHFrEF until 9-month follow-up (Fig. 1B and D; Table 1). Because of the lack of a specific dosage regimen for sacubitril/valsartan in MS-related cardiomyopathy, we used the recommended therapeutic scheme.^[11]^

**Table 1:**
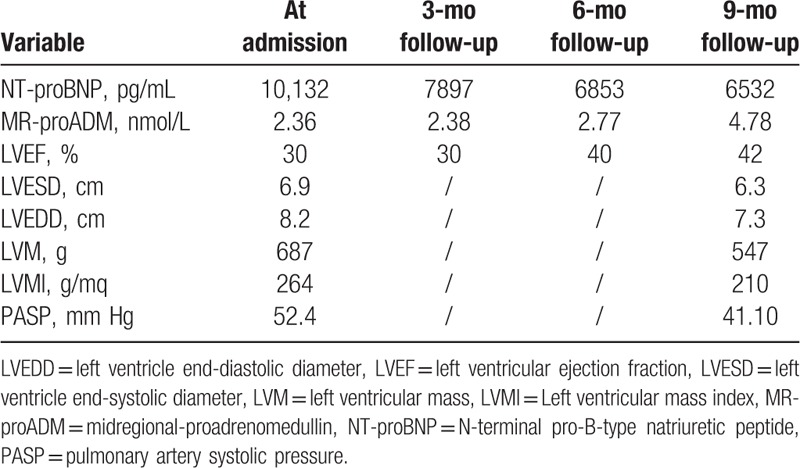
Laboratory variables and echocardiographic features at admission, 3-, 6-, and 9-month follow-up.

## 3 Discussion

We report the successful use of sacubitril/valsartan in a patient with MFS-related cardiomyopathy—NYHA III to IV class; stage C HFrEF with laboratoristic, echocardiographic, and quality of life improvement; and 9-month free survival from acute HF.

Before adding sacubitril/valsartan, patient presented recurrent ADHFrEF despite optimal medical therapy. To our knowledge, this is the first reported use of sacubitril/valsartan in a patient with HFrEF due to MFS.

**Figure 2:**
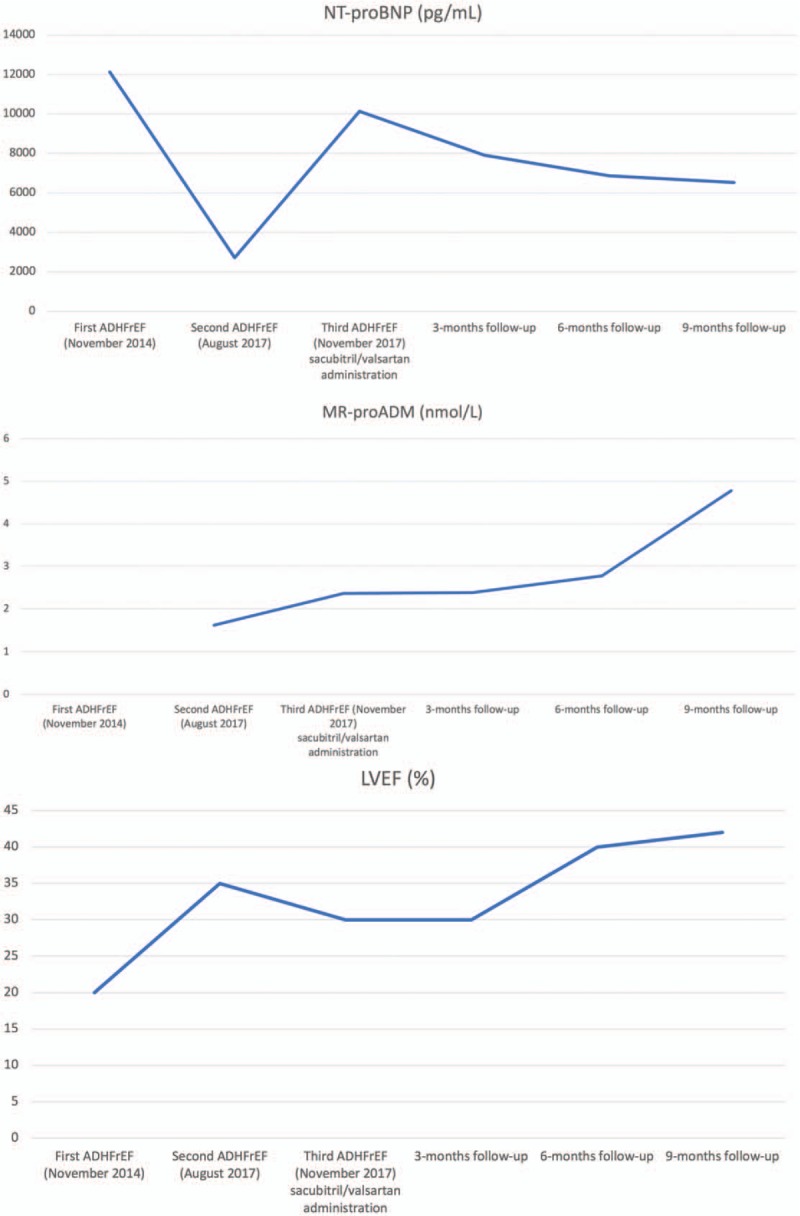
NT-proBNP (A), MR-proADM (B), and LVEF values (C) during the 3 ADHFrEF and 3-, 6-, and 9-month follow-up. ADHFrEF = acute decompensated heart failure with reduced ejection fraction, LVEF = left ventricular ejection fraction, MR-proADM = midregional-proadrenomedullin, NT-proBNP = N-terminal pro-B-type natriuretic peptide.

Acute HF determines BNP, ADM, bradykinin, and angiotensin II release through natriuretic peptide system and RAAS activation. These peptides are degraded by neprilysin reducing vasoconstriction, sodium retention, and maladaptive remodeling (Fig. 2).^[10]^ Therefore, although BNP and ADM—and its more stable and easily detectable MR-proADM^[12]^—reflect the effect of sacubitril/valsartan on the heart, NT-proBNP is not a neprilysin substrate and reflects hemodynamic status.

Sacubitril/valsartan effect are confirmed by improvement of echocardiographic features, LVEF increase of 5%, left ventricle end-systolic diameter, left ventricle end-diastolic diameter, left ventricular mass index reduction-free survival from HF (about 1–2 years), and quality of life.^[13,14]^ Furthermore, sacubitril/valsartan showed a higher efficacy, reduced rate of death from cardiovascular complications and hospitalization for HF ,a similar safety profile, and similar rate of adverse event (e.g., worsening renal function, hyperkalemia, symptomatic hypotension, and angioedema) compared with enalapril.^[6,9]^

In patients with MFS, *FBN1* mutation leads to fibrillin-1 deficiency causing activation of transforming growth factor-β (TGF-β) signaling pathways with improvement of collagen synthesis and elastic fiber disruption in cardiac and vessel wall connective tissue.^[15]^ This remodeling increases aortic stiffness and decreases vasoreactivity resulting in aortic dilatation, impaired diastolic ventricular relaxation, and hypertrophic remodeling.^[15]^

**Figure 3:**
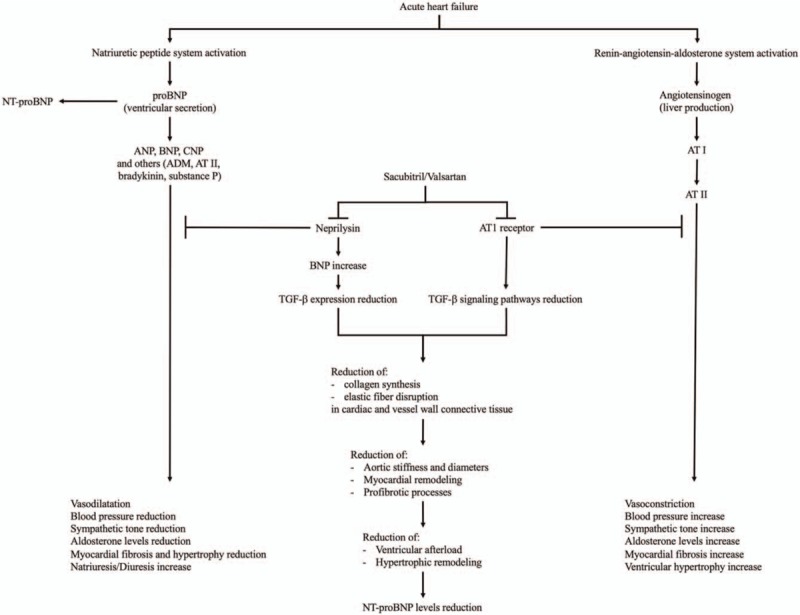
Acute heart failure determines natriuretic peptide and renin-angiotensin-aldosterone system activation with opposite effects from each other. Although sacubitril blocks neprilysin increasing natriuretic peptide system pathways, NT-proBNP is not a neprilysin substrate, valsartan inhibits angiotensin II type-1 (AT1) receptor reducing renin-angiotensin-aldosterone system effects. Furthermore, sacubitril/valsartan could be effective on MFS-related cardiovascular complications through TGF-β expression and signaling pathways reduction. ADM = adrenomedullin, ANP = atrial natriuretic peptide, AT = angiotensin, BNP = brain natriuretic peptide, CNP = C-type natriuretic peptide, MFS = Marfan syndrome, NT-proBNP = N-terminal pro-B-type natriuretic peptide, TGF-β = transforming growth factor-β.

Indirect inhibition of TGF-β, mediated by sacubitril-related neprilysin inhibition, could be hypothesized knowing that BNP opposes the expression of TGF-β-regulated genes,^[16]^ and that neprilysin inhibition by sacubitril causes an increase in BNP values (Fig. 3).^[17]^

Furthermore, effectiveness of angiotensin receptor blockers in cardiovascular complication of MFS was previously reported. AT1-receptor blockade showed capacity of reducing TGF-β signaling pathways along with other mechanisms responsible for reduction of cardiovascular disease progression (Fig. 3).^[18]^

After sacubitril/valsartan administration, patient showed persistent and progressive clinical and laboratory data (NT-proBNP reduction and MR-proADM increase), echocardiographic LVEF increase (42% vs 30%), left ventricle end-systolic diameter, left ventricle end-diastolic diameter, left ventricular mass and left ventricular mass index reduction, and quality of life improvement without new episodes of ADHFrEF until 9-month follow-up (Fig. 1B and D; Table 1).

To our knowledge, this is the first reported case of sacubitril/valsartan administration in patients with MFS-related cardiomyopathy. This case report highlights the beneficial effect of sacubitril/valsartan on pathophysiologic mechanisms of MFS-related cardiovascular complications and its possible administration in III–IV NYHA class, stage C reduced ejection fraction HF, once the hemodynamic stabilization was reached.

## Acknowledgments

Authors thank Valeriani Stefano for English language revision.

## Author contributions

**Conceptualization:** Silvia Spoto, Sebastiano Costantino, Massimo Ciccozzi, Silvia Angeletti.

**Data curation:** Silvia Spoto, Emanuele Valeriani, Luciana Locorriere, Giuseppina Beretta Anguissola, Angelo Lauria Pantano, Maria Caterina Bono, Sebastiano Costantino, Massimo Ciccozzi, Silvia Angeletti.

**Formal analysis:** Silvia Spoto, Luciana Locorriere, Giuseppina Beretta Anguissola, Angelo Lauria Pantano, Francesca Terracciani, Maria Caterina Bono, Sebastiano Costantino, Silvia Angeletti.

**Investigation:** Luciana Locorriere, Giuseppina Beretta Anguissola, Angelo Lauria Pantano, Francesca Terracciani, Sebastiano Costantino.

**Methodology:** Silvia Spoto, Emanuele Valeriani, Sebastiano Costantino, Silvia Angeletti.

**Supervision:** Sebastiano Costantino, Silvia Angeletti.

**Writing – original draft:** Silvia Spoto, Massimo Ciccozzi.

**Writing – review and editing:** Emanuele Valeriani, Sebastiano Costantino, Massimo Ciccozzi, Silvia Angeletti.
